# Rauscher virus-induced reticulum-cell sarcomas: Their growth in vitro and erythropoietic differentiation.

**DOI:** 10.1038/bjc.1977.27

**Published:** 1977-02

**Authors:** A. H. Fieldsteel, P. J. Dawson, F. A. Becker, C. Kurahara, C. Mitoma

## Abstract

A transplantable reticulum-cell sarcoma induced by Rauscher virus (RV) in (C57BL/6 X DBA/2)F1 (BDF1) mice was grown in tissue culture. Four separate cell lines were established, all of which grew predominantly in suspension. The doubling time of the cells from these cultures ranged from 17 to 32 h. Each culture continued to replicate RV, as indicated by the infectivity in newborn mice of all fluids tested up to the 75th passage. Since the morphological appearance of the cells in vitro was consistent with that of proerythroblasts, all cultures were tested for their ability to differentiate along the erythrocytic line under the influence of dimethylsulphoxide (DMSO). One of the cultures produced small quantities of haemoglobin independently of DMSO. Another was shown to produce haemoglobin, as well as to take up 59Fe and incorporate it into haem, only in the presence of DMSO. The 2 remaining cultures failed to produce haemoglobin, either spontaneously or in the presence of DMSO. Cells from each of the RV-induced cultures, when inoculated back into BDF1 mice, induced typical reticulum-cell sarcomas, without in vivo evidence of erythroid differentiation. In contrast, 2 morphologically identical but non-infectious cell lines derived from Friend virus-induced reticulum-cell sarcomas did not show erythroid differentiation in vivo or in vitro, either in the absence or presence of DMSO.


*Br. J. Cancer* (1977) **35**, 199

### RAUSCHER VIRUS-INDUCED RETICULUM-CELL SARCOMAS: THEIR GROWTH *IN VITRO* AND ERYTHROPOIETIC DIFFERENTIATION

A. H. FIELDSTEEL*, P. J. DAWSON†, F. A. BECKER*, C. KURAHARA* AND C. MITOMA*

*From the *Life Sciences Division, Stanford Research Institute, Menlo Park, California* 94025, *U.S.A., and the †Department of Pathology, University of Oregon Medical School, Portland, Oregon* 97201, *U.S.A.*

Received 10 August 1976 Accepted 11 October 1976

**Summary.—A transplantable reticulum-cell sarcoma induced by Rauscher virus (RV) in (C57BL/6 × DBA/2)$F_1$ ($BDF_1$) mice was grown in tissue culture. Four separate cell lines were established, all of which grew predominantly in suspension. The doubling time of the cells from these cultures ranged from 17 to 32 h. Each culture continued to replicate RV, as indicated by the infectivity in newborn mice of all fluids tested up to the 75th passage. Since the morphological appearance of the cells *in vitro* was consistent with that of proerythroblasts, all cultures were tested for their ability to differentiate along the erythrocytic line under the influence of dimethylsulphoxide (DMSO). One of the cultures produced small quantities of haemoglobin independently of DMSO. Another was shown to produce haemoglobin, as well as to take up $^{59}Fe$ and incorporate it into haem, only in the presence of DMSO. The 2 remaining cultures failed to produce haemoglobin, either spontaneously or in the presence of DMSO. Cells from each of the RV-induced cultures, when inoculated back into $BDF_1$ mice, induced typical reticulum-cell sarcomas, without *in vivo* evidence of erythroid differentiation. In contrast, 2 morphologically identical but non-infectious cell lines derived from Friend virus-induced reticulum-cell sarcomas did not show erythroid differentiation *in vivo* or *in vitro*, either in the absence or presence of DMSO.**

WE HAVE reported the induction of transplantable reticulum cell sarcomas (RCS) in (C57BL/6 × DBA/2)$F_1$ ($BDF_1$) and (C57BL/Ks × DBA/2)$F_1$ mice from the spleens of syngeneic donors infected with Rauscher virus (RV) (Dawson and Fieldsteel, 1974). These tumours were morphologically indistinguishable from the transplantable Friend virus (FV)-induced reticulum-cell sarcomas induced by us in BALB/c and $BDF_1$ mice (Fieldsteel, Dawson and Bostick, 1963; Fieldsteel, Dawson and Scholler, 1968). The FV-induced tumours cultured *in vitro* eventually lost their ability to replicate infectious FV (Fieldsteel, Dawson and Scholler, 1966; Fieldsteel, Kurahara and Dawson, 1969*b*), but they retained the FV genome, which could be retrieved with appropriate helpers (Fieldsteel, Kurahara and Dawson, 1969*c*; Fieldsteel, Dawson and Kurahara, 1971). The tumours induced by us differed from those induced in DBA/2 mice by other investigators in that the latter continued to relicate virus, differentiated along the erthyrocytic line, and were able to incorporate radioactive iron and synthesize haemoglobin (Friend *et al.*, 1971; Scher, Holland and Friend, 1971; Ostertag *et al.*, 1972). When dimethylsulphoxide (DMSO) was added to these cultures, the number of cells maturing along the erythrocytic line increased greatly, with proerythroblasts maturing to normoblasts. Increased synthesis of haemoglobin resulted. Erythroblastic proliferation is even more pronounced in Rauscher leukaemia than in Friend leukaemia. Pluznick, Sachs and Resnitzky (1966) have shown that the target cells for RV probably are erythroblasts. Yokoro and Thorell (1966) showed, by microspectrophotometric analysis of proliferating leukaemia cells in the livers of mice infected with RV, that there was a progressive sequence from cells containing little or no haemoglobin in the cytoplasm to almost fully haemoglobinized erythrocytes found in the peripheral blood at advanced stages of the disease. Brommer and Bentvelzen (1974) reported that the virus also invaded other types of haematopoietic cells, including stem cells. Because of the close relationship between FV and RV, and the similarity between the 2 tumours induced by these viruses, we attempted to cultivate the RV-induced RCS *in vitro*. This paper reports, for the first time, the successful cultivation of RV-induced reticulum-cell sarcomas, and the ability of one cell line to undergo erythroid differentiation.

### MATERIALS AND METHODS

*Animals.*—BALB/c mice were obtained from our own inbred colony. (C57BL/6 × DBA/2)$F_1$ ($BDF_1$), DBA/2 and Swiss mice were purchased from Simonsen Laboratories, Gilroy, California.

*Tumour.*—The tumour used to initiate the cell lines was induced by us in $BDF_1$ mice by s.c. inoculation of RV-infected spleen suspensions (Dawson and Fieldsteel, 1974). After 3 s.c. passages, the tumour was maintained by i.p. passage.

*Tissue cultures.*—The cultures were initiated from either the solid tumours harvested from the abdominal cavity after i.p. inoculation, or from the associated ascitic fluid, in a manner similar to that previously described by us for the cultivation of FV-induced RCS in tissue culture (Fieldsteel *et al.*, 1966; Fieldsteel *et al.*, 1969*b*). Briefly, the solid tumours were minced finely with iris scissors in Hanks' solution. The suspension, diluted to 5 ml, was taken up and expelled from a 10-ml glass syringe through an 18-gauge needle. This procedure was repeated several times using successively smaller needles. When the suspension passed easily through a 21-gauge needle, it was centrifuged lightly to remove all but the single cells. The suspension was then centrifuged at 500 *g* for 5 min to sediment the cells and resuspended in growth medium that consisted of RPMI 1630 supplemented with 10% inactivated foetal bovine serum (FBS). The ascitic fluids used for initiating the cultures were generally bloody, but they contained large numbers of tumour cells. These were separated from the red blood cells by centrifugation. The tumour cells were resuspended in the growth medium. The cells, 1 to 2 × $10^7$ viable, were suspended in 10 ml of medium and dispersed into either 8-oz prescription bottles or 120-ml milk dilution bottles. Incubation was at 34°C, which is our standard temperature for cultivating all cells. Initially, the cultures consisted of a combination of fibroblast-like cells growing on the glass, and small clusters of spherical cells growing in the suspension. Passage was made of only the suspended cells. When these were inoculated back into $BDF_1$ mice, they produced RCS after 19 to 64 days, depending on the number of cells inoculated.

In addition, 4 other suspension cell cultures were used in experiments reported here. Two of them (FVTCT-BALB and FVTCT-$BDF_1$) have previously been described in detail (Fieldsteel *et al.*, 1966; Fieldsteel *et al.*, 1969*b*); they were derived from RCS induced by FV in BALB/c and $BDF_1$ mice, respectively. They do not contain infectious FV, but they do contain the retrievable FV genome. The third cell line, GM-86 Clone 745 (also a FV-induced RCS) was initiated by Dr Charlotte Friend in DBA/2 mice (Friend and Haddad, 1960). It contains infectious FV, differentiates along the erythrocytic line and produces tumours in DBA/2 mice (Friend *et al.*, 1971; Scher *et al.*, 1971). The fourth cell line, received from A. D. Little, Inc., was derived from the chemically induced L1210 leukaemia of DBA/2 mice. All the cell lines were cultivated and passaged in RPMI 1630 medium plus 10% FBS, except GM-86 Clone 745, which was cultivated in Eagle's MEM plus 15% FBS.

*Tissue cell titration.*—Cells were harvested when at least 90% were viable as determined by trypan blue exclusion. The concentration of viable cells was adjusted to 5 × $10^6$/ml. Decimal dilutions were then made in culture medium and groups of 5 $BDF_1$ mice were inoculated, either s.c. or i.p., with 0·2 ml of these dilutions. Final concentrations of cells ranged from $10^1$ to $10^6$. After 60 days, the 50% tumour endpoint was calculated according to the Reed and Muench method.

*Virus determinations and titrations.*—To test for presence of RV in the cultures, fluids were removed after cultivation for 5 to 7 days. The cells were removed by several cycles of centrifugation followed by freezing to − 70°C and thawing at 37°C. The supernatant fluid (0·05 to 0·1 ml) was inoculated i.p. into litters of newborn BALB/c mice, which were then observed for the development of the splenomegaly typical of Rauscher disease; presence of the disease was confirmed histologically.

For the virus titrations, serial, ten-fold dilutions up to $10^{-4}$ were made of the cell-free tissue culture fluids and were inoculated into groups of newborn BALB/c mice. The mice were observed for 35 days; then the survivors were killed and all spleens, whether enlarged or not, were examined histologically. The $ID_{50}$ was then calculated according to the Reed and Muench method.

*Growth curves.*—The growth rates and doubling times of all cell lines were determined. Each culture was set up in duplicate in milk dilution bottles, and counts of total and viable cells were made daily, from the day the culture was initiated until the 4th day. Beyond that point, the culture was no longer in the logarithmic growth phase.

*Erythrocytic differentiation and tests for haemoglobin synthesis.*—To induce differentiation, unsterile Fisher's Certified A.C.S. grade DMSO was added to replicate cultures to a final concentration of 1% or 2%. DMSO was not added to control cultures, so that we could determine whether the cells differentiated in its absence. After incubation at 34°C for 6 days, the cultures were centrifuged at 500 *g* for 10 min, and the cells were resuspended in 10 ml of phosphate-buffered saline. This process was repeated twice and, after the final wash, the viable cells were counted. The cells were centrifuged again, resuspended in 2·5 ml of distilled $H_2O$, allowed to stand at room temperature for 10 min and then frozen and thawed 3 times. The suspensions were further subjected to sonication for 1 min, using a Bronwill Biosonik III with a " needle " probe. These lysates were then tested by the benzidine (Frankel and Reitman. 1973) and orthotolidine (Henry, 1964) tests for the presence of haemoglobin. In addition, histochemical staining was carried out on cells of 6-day-old cultures. The cells were centrifuged directly on to a standard microscope slide, by means of a Shandon-Elliot cytospin, and stained by Ralph's benzidine method for haemoglobin (LoBue *et al.*, 1963).

*Incorporation of $^{59}Fe$ into the tissue culture cells.*—The incorporation method used was that of Scher *et al.* (1971). The FBS to be used in the tissue culture was tested both for its total iron content and unsaturated iron-binding capacity. $^{59}FeSO_4$ was then added to the serum, in a quantity that did not exceed the iron-binding capacity. The culture medium was supplemented with 15% of the FBS containing $^{59}Fe$ (3 $\mu$Ci/culture). The cultures were incubated for 6 days at 34°C and then washed, counted and lysed as described above.

Each lysate was assayed for radioactivity, to determine the total iron taken up by the cells. Haem was then dissociated from globin, converted to haemin and extracted in cyclohexanone. The amount of $^{59}Fe$ incorporated into haem was determined by measuring radioactivity in the cyclohexanone extract. A Nuclear-Chicago gamma counter, Model 4224, was used to measure radioactivity.

### RESULTS

Four separate cell lines were established from the 4th, 11th and 19th *in vivo* passages of an RV-induced RCS from $BDF_1$ mice. Two of these originated from the same mouse at the 4th passage, one from a brei of solid tumour cells [RVTCT(124GG) S] and the other [RVTCT(124GG)A] from the ascitic cells. The line derived from the 11th passage was designated RVTCT (187GG) and the line from the 19th passage, RVTCT(133HH). Although the majority of cells grew as spherical cells in suspension, and only these were passaged, a varying number of fibroblast-like cells, less than 10%, grew on the glass with spherical cells apparently attached to them. No fibroblast-like cells have been identified in the culture isolated from the 11th *in vivo* passage (187GG).

During early passage, while the cultures were being established, the cells multiplied at a very slow rate. However, after 55 passages *in vitro*, RVTCT(124GG)A had a 20- to 21-h doubling time. At the 43rd tissue culture passage, the RVTCT (124GG)S had a doubling time of 19 h. At the 15th and 26th tissue culture passages of the RVTCT(187GG), the doubling times were 22 and 17 h respectively. The doubling time of RVTCT (133HH) at the 24th passage averaged 32 h.

Examination of cytospin smears, stained with Wright's stain, from all 3 RVTCT cultures as well as FVTCT-BALB, FVTCT-$BDF_1$, and Friend's GM-86 Clone 745 revealed very similar cell morphology in all cell lines. The cells varied in size from 12 to 28 $\mu$m in diameter. They were round and sharply circumscribed, with an occasional bleb at the cell surface. The periphery of the cytoplasm was homogeneous and deep blue, except immediately adjacent to the nucleus where it stained lighter and was vacuolated. The nuclei were likewise round and variable in size. The nuclear membrane was sharp, and the nuclear chromatin was coarsely stippled. One to 3 nucleoli were seen. Occasional giant and multinucleate forms were present. Mitoses were frequent. All cultures, especially GM-86 Clone 745, contained a population of smaller cells (approximately 7 to 8 $\mu$m in diameter). These had a more basophilic cytoplasm and condensed nuclear chromatin. A few such cells in GM-86 Clone 745 could be identified as haemoglobinized normoblasts, even without exposure to DMSO. The morphological appearance of the cells from both RVTCT and FVTCT cultures thus were consistent with that of proerythroblasts.

Cells from all the RVTCT lines, when inoculated back into adult $BDF_1$ mice, produced typical reticulum-cell sarcomas without evidence of erythroid differentiation. Cells from GM-86 Clone 745 produced identical tumours in DBA/2 mice. Following i.p. inoculation, the 50% tumour endpoint of RVTCT(124GG)A at the 21st passage was $6{\cdot}80 \times 10^3$, and that of RVTCT(187GG) at the 10th passage was $2{\cdot}08 \times 10^3$. The 50% tumour endpoint for RVTCT(133HH) has not yet been determined, but this line was shown to produce typical reticulum-cell sarcomas in $BDF_1$ mice after s.c. inoculation with $10^6$ cells.

Undiluted cell-free tissue culture fluids from various passages of all the cell lines were inoculated i.p. into newborn BALB/c mice to test for the presence of RV. The results (Table I) show persistence of RV in all the cell lines. In addition, fluids from RVTCT(187GG) were titrated at the 10th and 26th passages, and were found to contain $10^{2{\cdot}2}$ and $10^{3{\cdot}0}$ $ID_{50}$/ml of RV, respectively. Further, undiluted tissue culture fluid from GM-86 Clone 745 was tested in newborn Swiss mice and 6 out of 7 developed typical Friend disease.

The RVTCT cultures and the 2 FVTCT cultures were all tested for their ability to differentiate along erythrocytic lines under the influence of DMSO. The controls used were Friend's GM-86 Clone 745

TABLE I.—*Results of Tests for the Presence of Rauscher Virus in Tissue Culture Fluids of Rauscher Virus-induced Reticulum-cell Sarcomas Grown* in vitro (*RVTCT*)

| Tissue culture cell line | Number of passage levels tested | Range of passage levels tested | Cumulative results (no. with RD†/total) |
|---|---|---|---|
| RVTCT(124GG)A* | 11 | Original to 75 | 71/82 |
| RVTCT(124GG)S* | 8 | Original to 51 | 44/44 |
| RVTCT(187GG) | 15 | Original to 44 | 66/66 |
| RVTCT(133HH) | 4 | 1 to 31 | 36/36 |

* These cultures came from the same mouse. The " A " culture was started from ascitic fluid, and the " S " culture was started with a brei of solid tumour.

† Rauscher disease. The diagnosis was based on the presence of gross splenomegaly or, if this was absent, on the presence of typical disease microscopically.

culture, which was known to differentiate in the presence of DMSO (Scher, Preisler and Friend, 1973), and the L1210 culture which, as a lymphocytic leukaemia, was presumed not to differentiate.

All cultures were tested several times for their ability to replicate, as well as to differentiate, in the presence of 1% and 2% DMSO. The result of a typical experiment is given in Table II. The The 1% DMSO apparently had no toxic effect of the viability of any of the cell lines, compared with untreated control cultures, and showed only a slight inhibition of the L1210 line. However, the 2% concentration was inhibitory to all the cultures, and was especially toxic for the RVTCT(124GG) and RVTCT(187GG) cell lines. The lysates of all cultures were tested for the presence of haemoglobin, using the benzidine and orthotolidine tests and Ralph's staining technique. None of the cultures produced haemoglobin in the absence of DMSO, with the exception of RVTCT(124GG)A. In all the tests, this culture gave minimally positive results indicating the presence of small amounts of haemoglobin.

The only cultures that differentiated in the presence of 1% and 2% DMSO and showed a strong positive reaction for haemoglobin, were the known positive GM-86 Clone 745 and RVTCT(133HH). The remaining cultures, including RVTCT (124GG)A were negative in all 3 tests. It is of interest that RVTCT(133HH), when grown in the presence of DMSO at the 13th tissue culture passage gave only a questionably positive benzidine test. By the 18th passage it had become weakly positive, and by the 26th passage it was strongly positive and haemoglobinized normoblasts could be identified. However, the reaction was not as strong as that given by GM-86 Clone 745. Haemoglobin production by the latter was so striking that after centrifugation of the DMSO-treated cultures, the cell pellet appeared red. The only other culture in which this was subsequently observed was RVTCT(133HH), at the 31st passage, the highest passage thus far tested. The benzidine reaction at that passage was also comparable to that of the GM-86 Clone 745. In addition, the 31st passage RVTCT(133HH) culture, which was grown out in the absence of DMSO, showed a very occasional benzidine-positive cell indicating that differentiation was occurring in the absence of DMSO stimulation, albeit at a greatly reduced rate.

Measurements of the cellular uptake of iron and its incorporation into haem under the influence of 1% DMSO (Table III) confirmed the results of the other tests, and showed that only one of our cell lines, the RVTCT(133HH), was capable of differentiating along the erythrocytic line in a manner similar to that of GM-86 Clone 745. It is interesting to note that RVTCT(124GG)A took up more $^{59}Fe/10^{6}$ cells than any of the other cultures, either before or after the addition of DMSO, including those that differentiated. This culture, without DMSO, also contained as much $^{59}Fe$ in haem/$10^{6}$ cells (0·103 nmol) as the GM-86 Clone 745 in the presence of 1% DMSO. However, the percentage of $^{59}Fe$ in haem in the latter was 4·4 times greater than in the former. These data suggest that haemoglobin might also be present in the cells of RVTCT(124GG)A. Unlike the cultures of the control haemoglobin-producing FV-induced tumour (GM-86 Clone 745), the RVTCT(124GG)A culture was only weakly positive, when tested by the benzidine and *o*-tolidine staining of whole or lysed cells. The cyclohexanone-extractable $^{59}Fe$ in this culture could represent either the presence of an uncharacterized haemoglobin-like material that did not react with the 2 stains employed or, less likely, contamination of the extract with inorganic $^{59}Fe$. In any event, this iron uptake was not stimulated by DMSO and is not interpreted as showing erythroid differentiation.

### DISCUSSION

Our previous success in establishing non-producer cell lines from FV-induced

TABLE II.—*Effect of DMSO on Cell Replication and Haemoglobin Synthesis of Friend and Rauscher Virus-induced Tumours Grown in Tissue Culture*

| Tissue culture cell line* | No DMSO | | | 1% DMSO‡ | | | 2% DMSO‡ | | |
|---|---|---|---|---|---|---|---|---|---|
| | Total cells ($\times 10^6$) | % viable | Haemoglobin tests† | Total cells ($\times 10^6$) | % viable | Haemoglobin tests† | Total cells ($\times 10^6$) | % viable | Haemoglobin tests† |
| FVCTC-BALB | 19·9 | 47 | — | 20·3 | 63 | — | 5·65 | 82 | — |
| FVTCT-BDF$_1$ | 22·4 | 89 | — | 28·2 | 94 | — | 4·50 | 89 | — |
| RVTCT(124GG)A | 6·15 | 52 | ± | 7·2 | 64 | — | 0·55 | 14 | — |
| RVTCT(187GG) | 11·7 | 62 | — | 4·9 | 69 | — | 0·43 | 0 | — |
| RVTCT(133HH) | 20·3 | 85 | — | 13·6 | 84 | + | 1·10 | 43 | + |
| GM-86 Clone 745 | 8·40 | 90 | — | 15·1 | 81 | + | 1·00 | 79 | + |
| L1210 | 29·5 | 96 | — | 10·3 | 92 | — | 1·08 | 90 | — |

* The number of cells added to each culture in 10 ml averaged $1{\cdot}06 \times 10^6$, and ranged between $8 \times 10^5$ and $2 \times 10^6$, depending on the known characteristic of each culture. Incubation was at 34°C for 6 days.

† Results of benzidine, orthotolidine and Ralph's stain, all of which gave similar results.

‡ Final concentration in 10 ml of culture medium added at initiation of culture.

TABLE III.—*Comparison of Haemoglobin Synthesis as Measured by the Uptake of $^{59}Fe$ in Various Friend and Rauscher Virus-induced Tumours in Tissue Culture*

| Tissue culture cell line | DMSO (%) | No. of cells in culture ($\times 10^6$) | nmol $^{59}Fe/10^6$ cells | nmol haem $^{59}Fe/10^6$ cells | % of $^{59}Fe$ in haem |
|---|---|---|---|---|---|
| (A) FVTCT-BALB | 0 | 19·9 | 0·539 | 0·011 | 2·04 |
| | 1 | 22·3 | 0·589 | 0·008 | 1·36 |
| (B) FVTCT-BDF$_1$ | 0 | 22·4 | 0·561 | 0·012 | 2·14 |
| | 1 | 28·2 | 0·448 | 0·006 | 1·34 |
| (C) RVTCT(124GG)A | 0 | 6·15 | 1·91 | 0·103 | 5·39 |
| | 1 | 7·20 | 1·72 | 0·073 | 4·24 |
| (D) RVTCT(187GG) | 0 | 11·7 | 0·895 | 0·017 | 1·90 |
| | 1 | 4·90 | 1·42 | 0·038 | 2·68 |
| (E) RVTCT(133HH) | 0 | 20·3 | 0·492 | 0·021 | 4·27 |
| | 1 | 13·6 | 0·600 | 0·144 | 24·0 |
| (F) GM-86 Clone 745 | 0 | 8·40 | 0·569 | 0·011 | 1·93 |
| | 1 | 15·1 | 0·438 | 0·103 | 23·5 |
| (G) L1210 | 0 | 29·5 | 0·140 | 0·002 | 1·43 |
| | 1 | 10·3 | 0·333 | — | — |

Cultures A and B are FV-induced and contain no infectious virus.
Cultures C, D and E are RV-induced and do contain infectious virus.
Culture F is FV-induced, contains infectious FV, and was initiated by C. Friend.
Culture G is a non-virally induced leukaemia of DBA/2 mice.

RCS (Fieldsteel *et al.*, 1966; Fieldsteel *et al.*, 1969*b*) led to similar attempts with an RV-induced RCS, particularly since the 2 viruses share a number of characteristics. However, although the viruses are closely related immunologically, they do not appear to be identical (Fink, Rauscher and Chirigos, 1966). We had previously isolated the lymphatic leukaemia virus (LLV-R) associated with RV (Fieldsteel, Dawson and Kurahara, 1969*a*) indicating the possibility that RV, like FV, was defective. If a non-producer RVTCT that contains the retrievable RV genome could be established, it would then be possible to further explore the differences between FV and RV and to determine if the differences were related to their respective helper viruses.

We were able to establish in continuous culture separate cells lines from an RCS induced in BDF$_1$ mice by RV. The cells of these cultures grew in suspension, as did the previously established cell lines from FV. The RVTCT cells differed from the FVTCT cells in that a small percentage of cells in 3 of the former were fibroblast-like and grew on the glass. All attempts to rid the cultures of these cells failed, as did attempts to obtain pure cultures of fibroblast-like cells, free from the spherical suspended cells.

All the cultures replicated RV at the time of initiation and, to date, none of them show any indication of lessened viral activity. When inoculated back into syngeneic BDF$_1$ mice, all the cultures induced tumours identical to those induced by the transplantable RV-induced tumour from which they arose. Although we have termed these tumours " reticulum-cell sarcomas " to distinguish them from the LLV-R-induced lymphocytic leukaemias, it seems clear that they are, in reality, erythroid precursors. It is interesting that in haematoxylin-and-eosin-stained paraffin sections, these tumours give no hint of erythroid differentiation, and are morphologically similar to tumours that in the past have been called reticulum-cell sarcomas. It is now generally recognized that most of the tumours so designated are not derived from histiocytes, but are actually composed of transformed lymphocytes (Braylan, Jaffe and Berard, 1975). Apparently, under the appropriate circumstances, erythroid precursors can take on similar morphological features.

None of the tumours examined by us, *i.e.* the 3 FV-induced tumours (FVTCT-

BALB, FVTCT-BDF$_1$, and GM-86 Clone 745) and the 4 RV-induced tumours, could be distinguished microscopically, yet they differed in several important biological characteristics. The 2 FVTCT tumours did not contain infectious FV, and they could not be induced to differentiate by DMSO. The GM-86 Clone 745 initiated by Dr Friend, contained infectious FV and readily differentiated along the erythrocytic pathway under the influence of DMSO. The 4 RVTCT tumours differed from the others in that they all contained infectious virus, but only one of them, RVTCT(133HH), could be stimulated to differentiate with DMSO. In that respect, as well as morphologically, the latter could not be distinguished from the FV-induced GM-86 Clone 745. However, they could be distinguished by the fact that RVTCT(133HH) produced tumours in BDF$_1$ mice, but not in DBA/2 mice, the strain of origin of GM-86 Clone 745.

Whether infectious RV or FV is required for differentiation was not determined. Because the 2 lines that differentiated contained infectious virus, and neither of the non-infectious lines could be induced to differentiate, it is possible that virus replication is necessary for haemoglobin production. However, Swetly and Ostertag (1974) used interferon to inhibit FV synthesis in their FV-transformed cell line, and simultaneously showed that the ability of the cells to differentiate and synthesize haemoglobin in the presence of DMSO was unaffected. They then concluded that the release of FV was not required for *in vitro* erythroid differentiation of those cells.

Of primary importance, however, is that the cells of the so-called reticulum-cell sarcomas induced by both FV and RV are probably of the same origin. That these cells are erythroid in nature is indicated by the fact that tissue culture cell lines, derived from RCS induced by both viruses, can be induced by DMSO to differentiate into haemoglobinized cells. That some of the tissue culture cell lines derived from RCS induced by both viruses cannot be induced to differentiate is of equal interest. This could indicate that although these non-differentiating cell lines also originate from erythroid cells, they might be derived from precursors more closely related to stem cells. Under those circumstances, the cells are possibly too primitive to be induced to differentiate under the influence of DMSO.

This investigation was supported by USPHS Grants CA-07868 and CA-15072 from the National Cancer Institute.

### REFERENCES

BRAYLAN, R. C., JAFFE, E. S. & BERARD, C. W. (1975) Malignant Lymphomas: Current Classification and New Observations. In *Pathol. Ann.*, Vol. 10, Ed. S. C. Sommers. Englewood Cliffs: Prentice-Hall, Inc., p. 213.

BROMMER, E. J. P. & BENTVELZEN, P. (1974) The Haematopoietic Stem Cell in Rauscher Virus-induced Erythroblastosis of BALB/c Mice. *Eur. J. Cancer*, **10**, 827.

DAWSON, P. J. & FIELDSTEEL, A. H. (1974) Reticulum Cell Sarcomas Induced in Mice by Rauscher Virus. *J. natn. Cancer Inst.*, **52**, 1805.

FIELDSTEEL, A. H., DAWSON, P. J. & BOSTICK, W. L. (1963) Viral Studies on Generalized Friend Disease and a Tumor Variant in BALB/c and Related Hybrid Mice. *Cancer Res.*, **23**, 355.

FIELDSTEEL, A. H., DAWSON, P. J. & KURAHARA, C. (1969*a*) Induction of Lymphatic Leukaemia in BALB/c Mice from the Original Isolate of Rauscher Virus. *Br. J. Cancer*, **23**, 806.

FIELDSTEEL, A. H., DAWSON, P. J. & KURAHARA, C. (1971) *In vivo* and *In vitro* Recovery of Defective Friend Virus by Various Leukemia Viruses. *Int. J. Cancer*, **8**, 304.

FIELDSTEEL, A. H., DAWSON, P. J. & SCHOLLER, J. (1966) Virus-free Friend Virus-induced Tumors: *In vitro* and *In vivo* Characteristics. *J. natn. Cancer Inst.*, **36**, 71.

FIELDSTEEL, A. H., DAWSON, P. J. & SCHOLLER, J. (1968) Friend Disease and a Tumor Variant in Hybrid (BDF$_1$) Mice. *Proc. Soc. exp. Biol. Med.*, **127**, 614.

FIELDSTEEL, A. H., KURAHARA, C. & DAWSON, P. J. (1969*b*) Friend Virus-induced Reticulum Cell Sarcomas Grown *In vitro*: Further Evidence for the Absence of Friend Virus. *Cancer Res.*, **29**, 1846.

FIELDSTEEL, A. H., KURAHARA, C. & DAWSON, P. J. (1969*c*) Moloney Leukaemia Virus as a Helper in Retrieving Friend Virus from a Non-infectious Reticulum Cell Sarcoma. *Nature, Lond.*, **223**, 1274.

FINK, M. A., RAUSCHER, F. J. & CHIRIGOS, M. (1966) Some Immune Reactions of Murine Leukemia Viruses Demonstrated within a Completely Isologous System. In *Viruses Inducing Cancer*—

*Implication for Therapy*. Ed. W. J. Burdette. Salt Lake City: University of Utah Press. p. 25.

FRANKEL, S. & REITMAN, S. (1963) *Gradwohl's Clinical Laboratory Methods and Diagnosis*, Vol. 2. 6th Ed. St. Louis: The C. V. Mosby Co. p. 1835.

FRIEND, C. & HADDAD, J. R. (1960) Tumor Formation with Transplants of Spleen or Liver from Mice with Virus-induced Leukemia. *J. natn. Cancer Inst.*, **25**, 1279.

FRIEND, C., SCHER, W., HOLLAND, J. G. & SATO, T. (1971) Hemoglobin Synthesis in Murine Virus-induced Leukemic Cells *In vitro*: Stimulation of Erythroid Differentiation by Dimethyl Sulfoxide. *Proc. natn. Acad. Sci U.S.A.*, **68**, 378.

HENRY, R. J. (1964) In *Clinical Chemistry: Principles and Techniques*. 1st Ed. New York: Harper & Rowe. p. 784.

LOBUE, J., DORNFEST, B. S., GORDON, A. S., HURST, J. & QUASTLER, H. (1963) Marrow Distribution in Rat Femurs Determined by Cell Enumeration and $Fe^{59}$ Labeling. *Proc. Soc. exp. Biol. Med.*, **112**, 1058.

OSTERTAG, W., MELDERIS, H., STEINHELDER, G., KLUGE, N. & DUBE, S. (1972) Synthesis of Mouse Haemoglobin and Globin mRNA in Leukaemic Cell Cultures. *Nature, New Biol.*, **239**, 231.

PLUZNIK, D. H., SACHS, L. & RESNITZKY, P. (1966) The Mechanism of Leukemogenesis by the Rauscher Leukemia Virus. *Natn. Cancer Inst. Monogr.*, **22**, 3.

SCHER, W., HOLLAND, J. G. & FRIEND, C. (1971) Hemoglobin Synthesis in Murine Virus-induced Leukemic Cells *In vitro*. I. Partial Purification and Identification of Hemoglobins. *Blood*, **37**, 428.

SCHER, W., PREISLER, H. D. & FRIEND, C. (1973) Hemoglobin Synthesis in Murine Virus-induced Leukemic Cells *In vitro*. III. Effects of 5-Bromo-2′-Deoxyuridine, Dimethylformamide and Dimethylsulfoxide. *J. Cell. Physiol.*, **81**, 63.

SWETLY, F. & OSTERTAG, W. (1974) Friend Virus Release and Induction of Haemoglobin Synthesis in Erythroleukaemic Cells Respond Differently to Interferon. *Nature, Lond.*, **251**, 642.

YOKORO, Y. & THORELL, B. (1966) Cytology and Pathogenesis of Rauscher Virus Disease in Splenectomized Mice. *Cancer Res.*, **26**, 536.


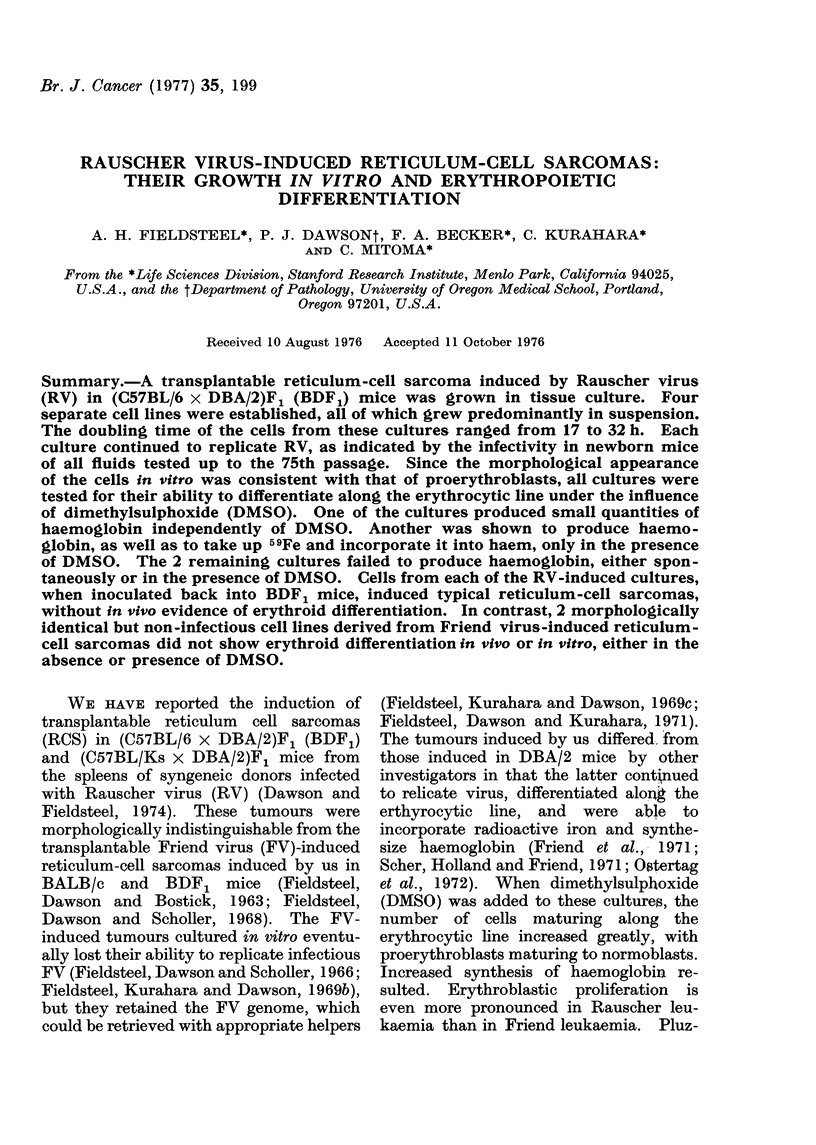

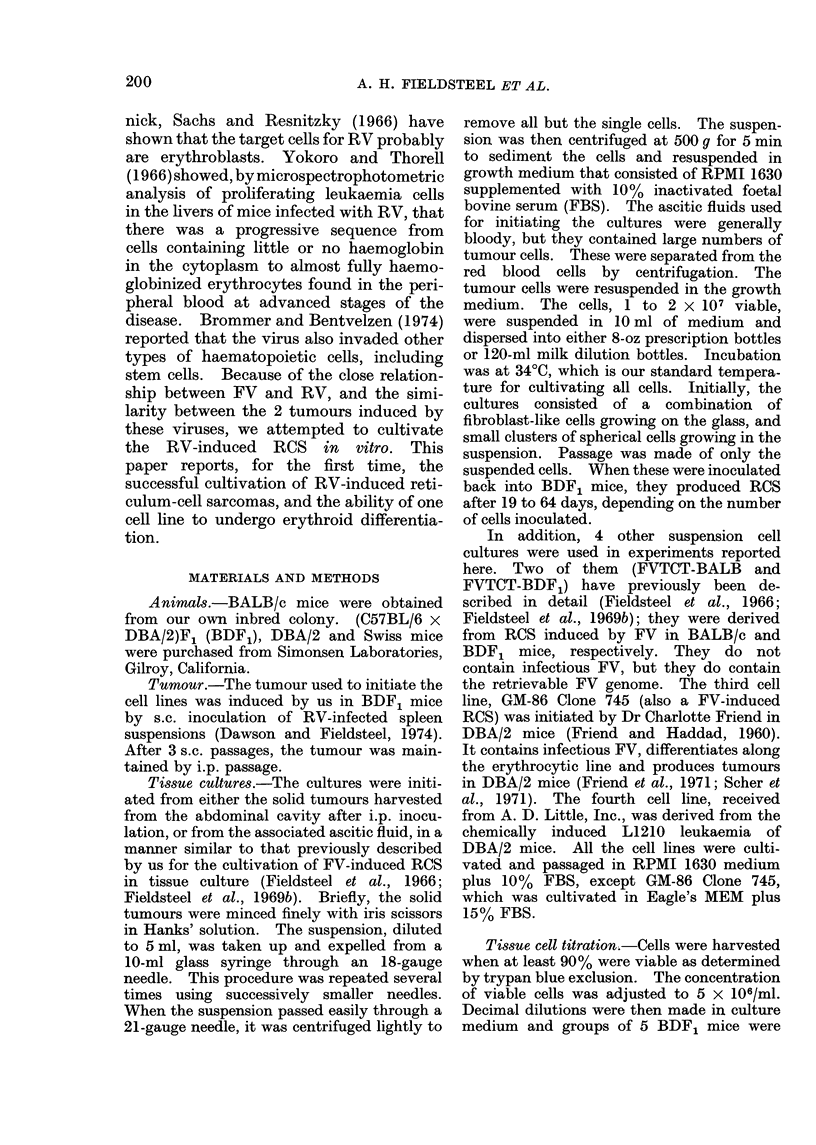

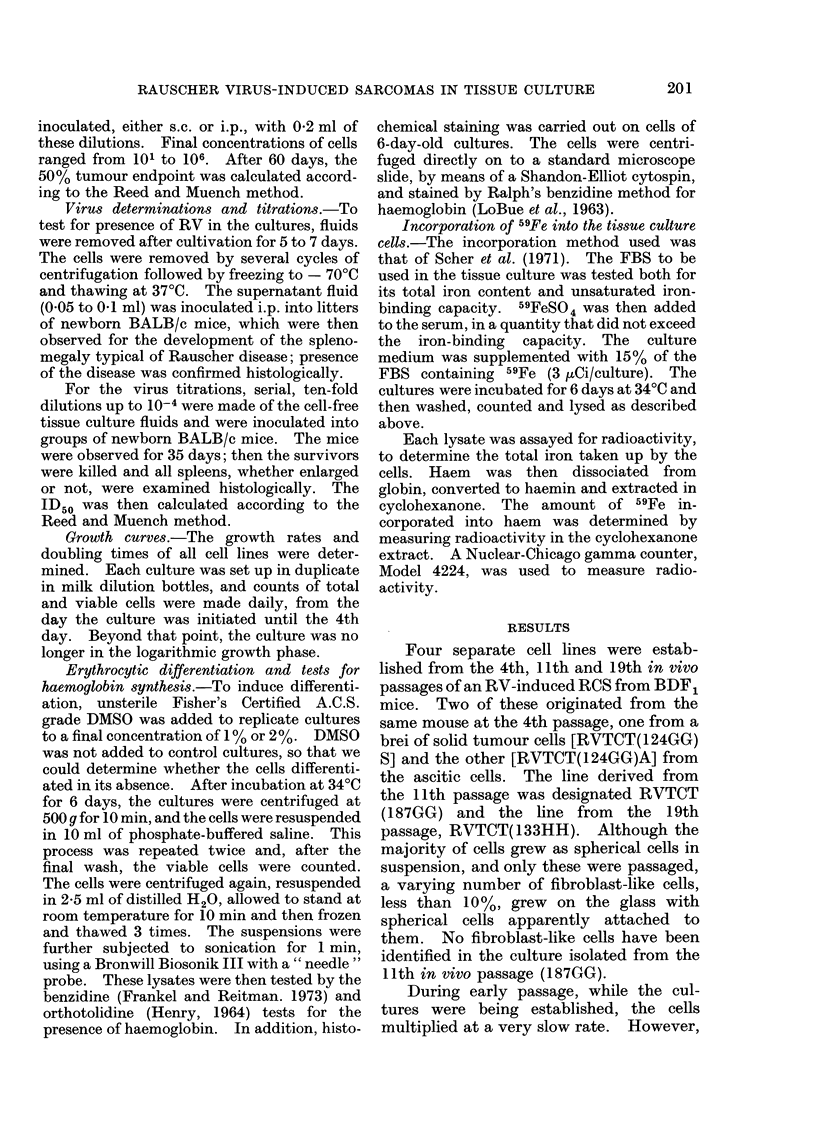

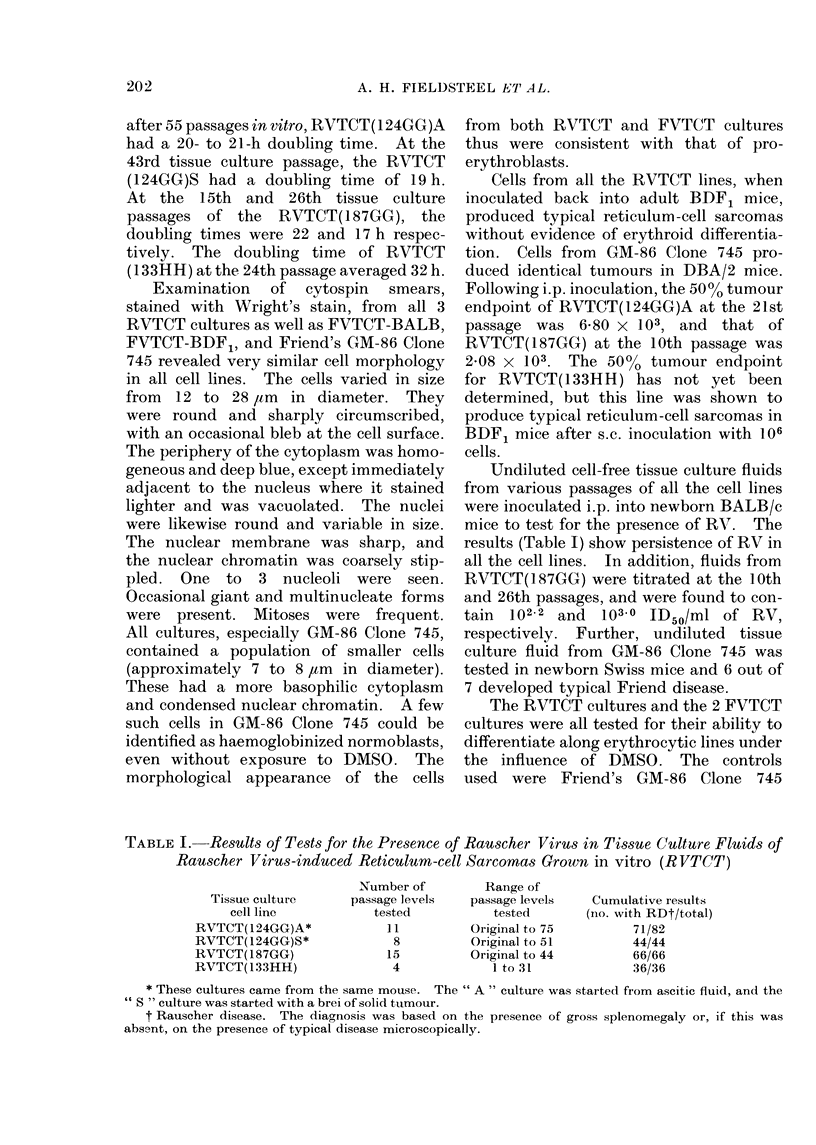

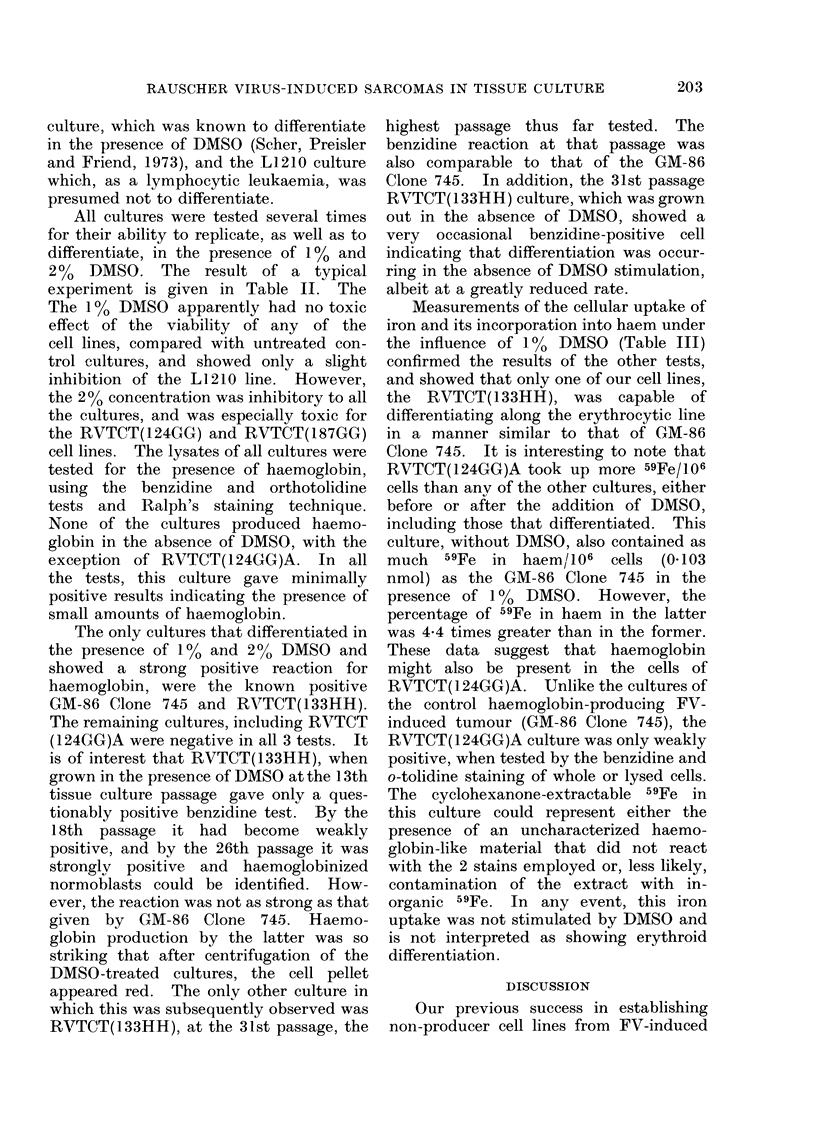

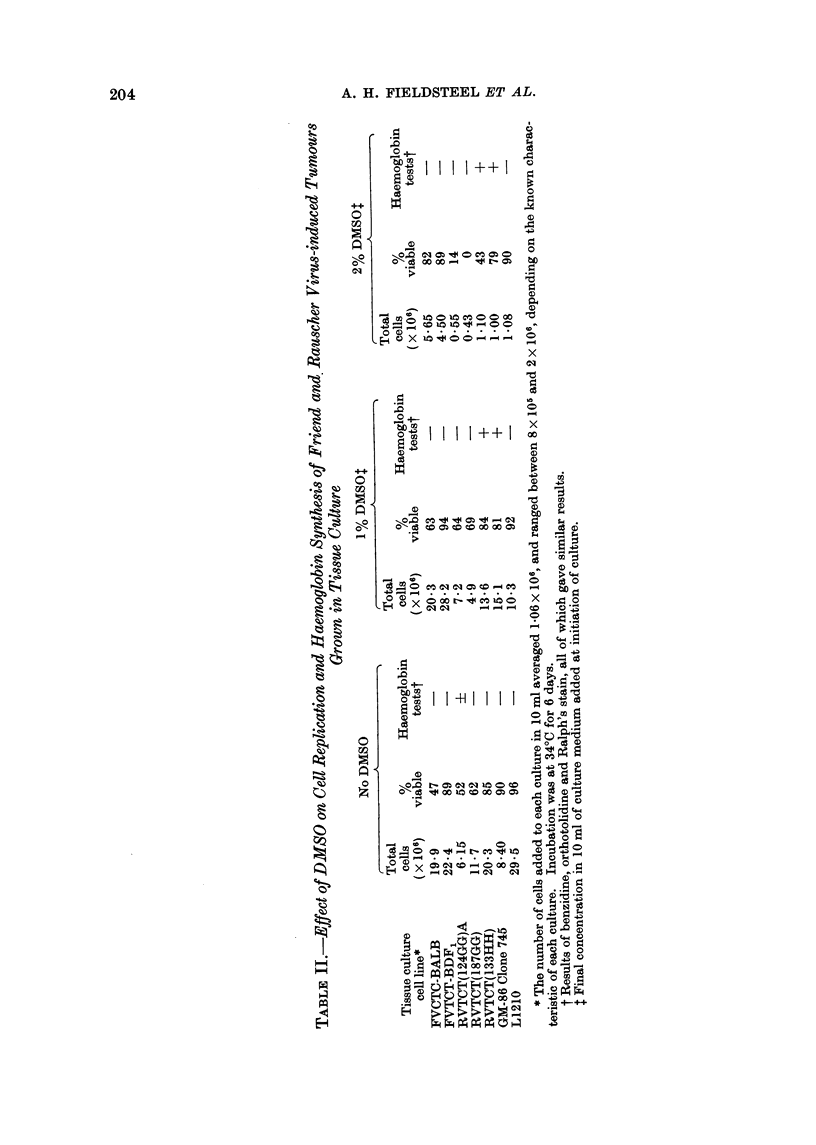

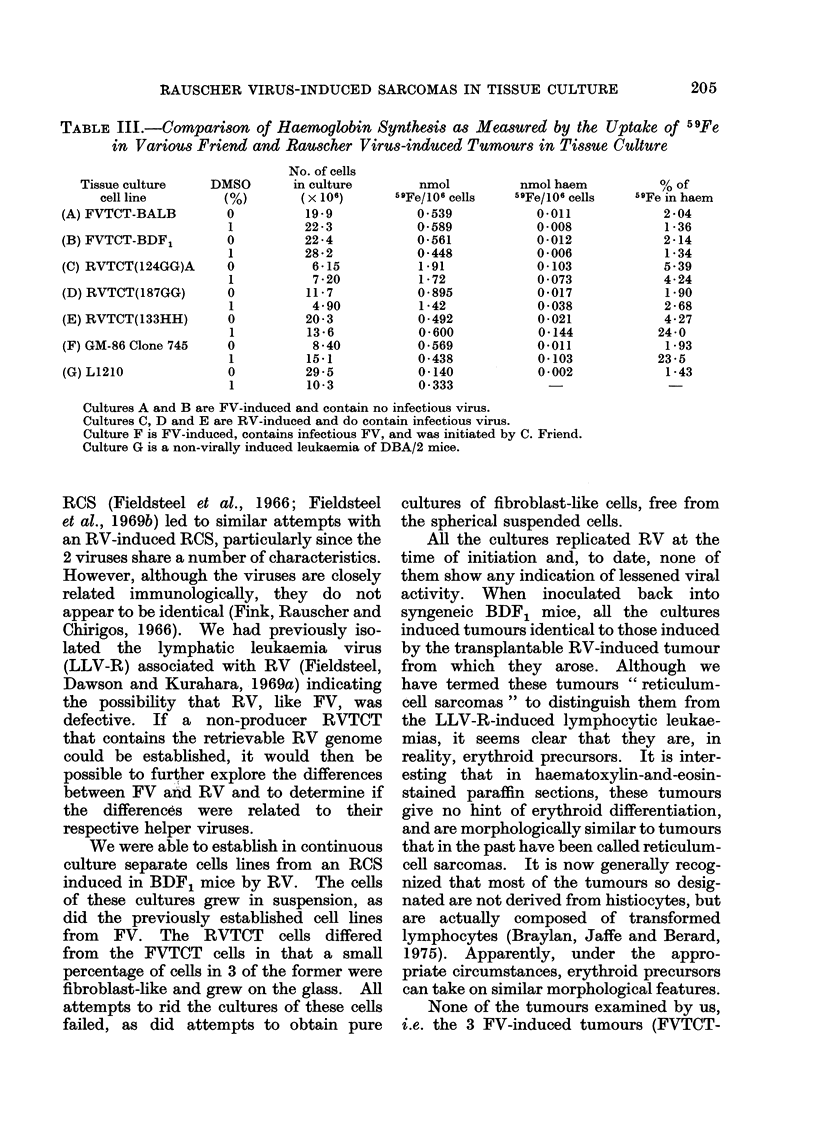

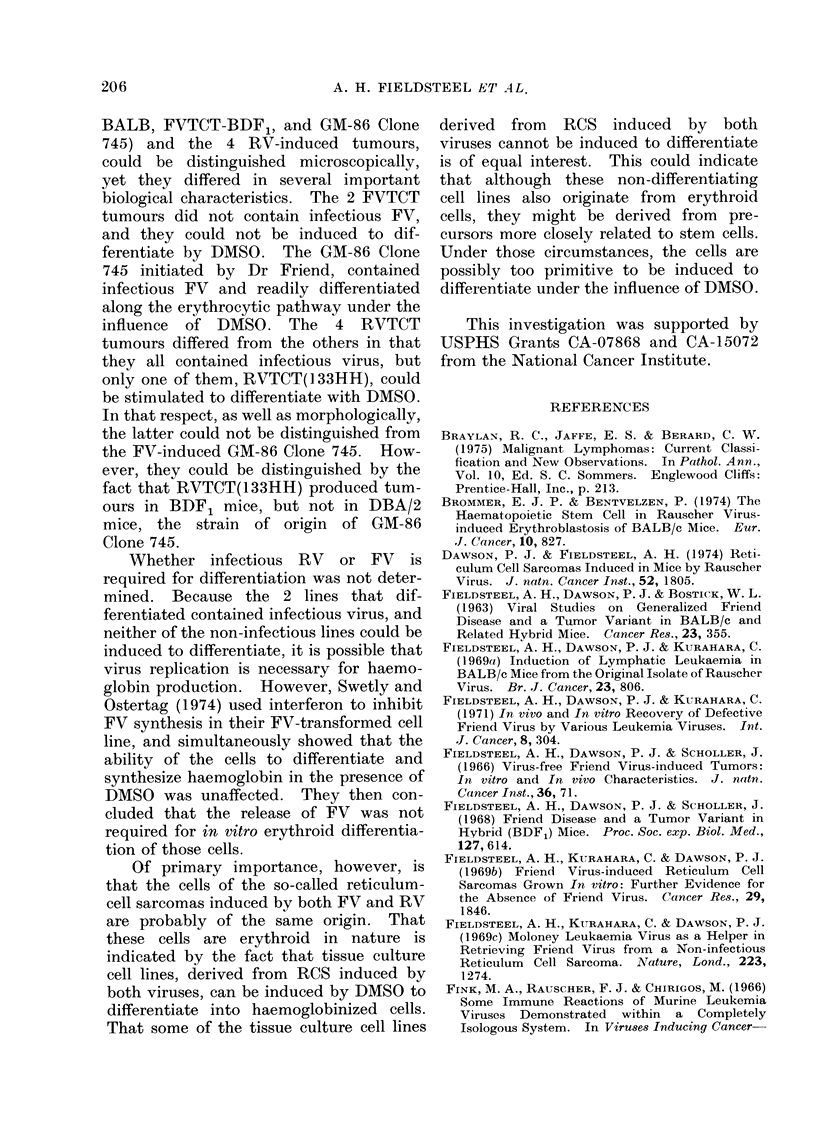

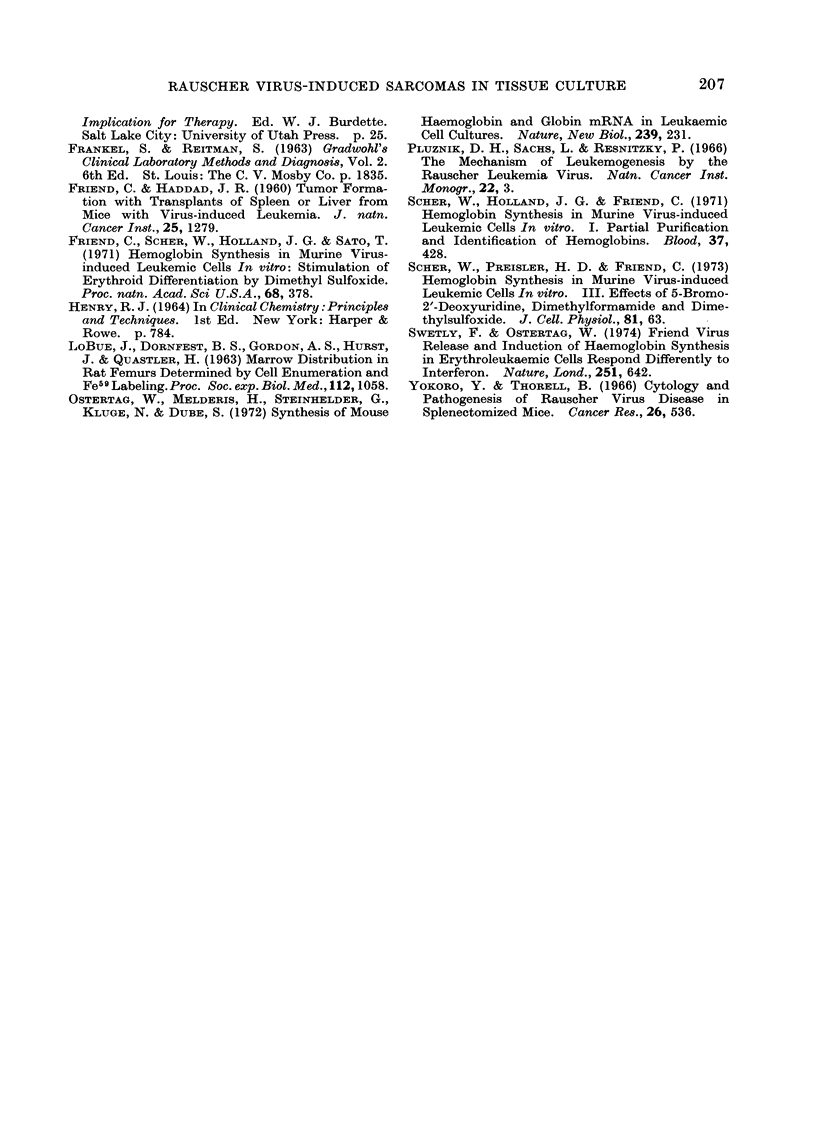


## References

[OCR_00974] Braylan R. C., Jaffe E. S., Berard C. W. (1975). Malignant lymphomas: current classification and new observations.. Pathol Annu.

[OCR_00981] Brommer E. J., Bentvelzen P. (1974). The haematopoietic stem cell in Rauscher virus-induced erythroblastosis of BALB/c mice.. Eur J Cancer.

[OCR_00987] Dawson P. J., Fieldsteel A. H. (1974). Reticulum cell sarcomas induced in mice by Rauscher virus.. J Natl Cancer Inst.

[OCR_00992] FIELDSTEEL A. H., DAWSON P. J., BOSTICK W. L. (1963). Viral studies on generalized Friend disease and a tumor variant in BALB/c and related hybrid mice.. Cancer Res.

[OCR_01050] FRIEND C., HADDAD J. R. (1960). Tumor formation with transplants of spleen or liver from mice with virus-induced leukemia.. J Natl Cancer Inst.

[OCR_01004] Fieldsteel A. H., Dawson P. J., Kurahara C. (1971). In vivo and in vitro recovery of defective Friend virus by various leukemia viruses.. Int J Cancer.

[OCR_01016] Fieldsteel A. H., Dawson P. J., Scholler J. (1968). Friend disease and a tumor variant in hybrid (BDF1) mice.. Proc Soc Exp Biol Med.

[OCR_01010] Fieldsteel A. H., Dawson P. J., Scholler J. (1966). Virus-free Friend virus-induced tumor: in vitro and in vivo characteristics.. J Natl Cancer Inst.

[OCR_01029] Fieldsteel A. H., Kurahara C. (1969). Moloney leukaemia virus as a helper in retrieving Friend virus from a non-infectious reticulum cell sarcoma.. Nature.

[OCR_01056] Friend C., Scher W., Holland J. G., Sato T. (1971). Hemoglobin synthesis in murine virus-induced leukemic cells in vitro: stimulation of erythroid differentiation by dimethyl sulfoxide.. Proc Natl Acad Sci U S A.

[OCR_01068] LOBUE J., DORNFEST B. S., GORDON A. S., HURST J., QUASTLER H. (1963). Marrow distribution in rat femurs determined by cell enumeration and Fe59 labeling.. Proc Soc Exp Biol Med.

[OCR_01073] Ostertag W., Melderis H., Steinheider G., Kluge N., Dube S. (1972). Synthesis of mouse haemoglobin and globin mRNA in leukaemic cell cultures.. Nat New Biol.

[OCR_01080] Pluznik D. H., Sachs L., Resnitzky P. (1966). The mechanism of leukemogenesis by the Rauscher leukemia virus.. Natl Cancer Inst Monogr.

[OCR_01086] Scher W., Holland J. G., Friend C. (1971). Hemoglobin synthesis in murine virus-induced leukemic cells in vitro. I. Partial purification and identification of hemoglobins.. Blood.

[OCR_01100] Swetly P., Ostertag W. (1974). Friend virus release and induction of haemoglobin synthesis in erythroleukaemic cells respond differently to interferon.. Nature.

[OCR_01106] Yokoro K., Thorell B. (1966). Cytology and pathogenesis of Rauscher virus disease in splenectomized mice.. Cancer Res.

